# Exploring Precise Medication Strategies for OSCC Based on Single-Cell Transcriptome Analysis from a Dynamic Perspective

**DOI:** 10.3390/cancers14194801

**Published:** 2022-09-30

**Authors:** Qingkang Meng, Feng Wu, Guoqi Li, Fei Xu, Lei Liu, Denan Zhang, Yangxu Lu, Hongbo Xie, Xiujie Chen

**Affiliations:** 1 Department of Pharmacogenomics, College of Bioinformatics Science and Technology, Harbin Medical University, Harbin 150081, China; 2 Department of Orthopedics, The Second Affiliated Hospital of Harbin Medical University, Harbin 150086, China

**Keywords:** single-cell RNA-Seq, oral squamous cell carcinoma, cell trajectory inference, precise medication, drug discovery

## Abstract

**Simple Summary:**

At the time of diagnosis, most oral squamous cell carcinoma (OSCC) patients are in the middle or advanced stages, and advanced patients usually have poor prognosis after traditional therapy. One of the primary causes has been demonstrated to be heterogeneity. However, most of the current studies on tumor heterogeneity are static, while the development of cancer is dynamic. Thus, understanding the tumor development process from a dynamic perspective is deeply necessary. Here, we combined static and dynamic analysis based on single-cell RNA-Seq data to comprehensively dissect the complex heterogeneity and evolutionary process of OSCC. We pioneered the concept of pseudo-time score, which is closely related to patient’s prognosis. Finally, we identified candidate drugs and proposed precision medication strategies to control OSCC in two respects: treatment and blocking. Our findings offer new insights for clinical practice and could help improve the treatment of advanced OSCC.

**Abstract:**

At present, most patients with oral squamous cell carcinoma (OSCC) are in the middle or advanced stages at the time of diagnosis. Advanced OSCC patients have a poor prognosis after traditional therapy, and the complex heterogeneity of OSCC has been proven to be one of the main reasons. Single-cell sequencing technology provides a powerful tool for dissecting the heterogeneity of cancer. However, most of the current studies at the single-cell level are static, while the development of cancer is a dynamic process. Thus, understanding the development of cancer from a dynamic perspective and formulating corresponding therapeutic measures for achieving precise treatment are highly necessary, and this is also one of the main study directions in the field of oncology. In this study, we combined the static and dynamic analysis methods based on single-cell RNA-Seq data to comprehensively dissect the complex heterogeneity and evolutionary process of OSCC. Subsequently, for clinical practice, we revealed the association between cancer heterogeneity and the prognosis of patients. More importantly, we pioneered the concept of pseudo-time score of patients, and we quantified the levels of heterogeneity based on the dynamic development process to evaluate the relationship between the score and the survival status at the same stage, finding that it is closely related to the prognostic status. The pseudo-time score of patients could not only reflect the tumor status of patients but also be used as an indicator of the effects of drugs on the patients so that the medication strategy can be adjusted on time. Finally, we identified candidate drugs and proposed precision medication strategies to control the condition of OSCC in two respects: treatment and blocking.

## 1. Introduction

Oral squamous cell carcinoma (OSCC) is the most common type of head and neck squamous cell carcinoma (HNSC), accounting for approximately 95% of cases [1]. At present, the clinical classification and treatment of OSCC are mainly based on the TNM staging system of the American Joint Committee on Cancer (AJCC) and the International Union for Cancer Control (UICC). In general, patients in the early stages (i.e., stages I and II)—approximately 30–40% of the patients diagnosed—have small tumors without significant lymph node involvement. Surgery and radiation therapy can provide effective tumor control and improve long-term survival in approximately 70–90% of early stage patients [2]. For patients with advanced stages (i.e., stages III and IV), which are characterized by varying degrees of surrounding tissue invasion, lymph node involvement, and metastatic spread, it is difficult to eliminate or kill tumors completely via surgery or radiotherapy. Although systematic treatment with drugs (e.g., platinum, taxanes, antifolates, cetuximab, etc.) can be conducted for the remission of recurrence and metastasis [2], more than 65% of these patients have a poor prognosis due to the significant heterogeneity, which appears not only between individuals but also within the same individual or even the same tissue—that is, intratumoral heterogeneity [3]. Therefore, conquering advanced OSCC has become an urgent problem to be solved in the field of HNSC treatment. Although many studies have been carried out successively, and some progress has been made in the diagnosis and treatment of OSCC, most advanced patients will still experience recurrence or metastasis (or both) [2]. Further study of the heterogeneity of advanced OSCC and, accordingly, exploration of improved therapeutic strategies to perform more precise treatment and increase the cure rate is highly necessary.

In recent years, the rapid development of single-cell sequencing technology has provided a powerful tool for basic cancer research. Relative to traditional bulk sequencing methods, single-cell sequencing takes a single cell as the basic unit and, therefore, provides much better insight into the heterogeneity between cells. The methods based on single-cell sequencing bring new opportunities to dissect the intratumoral heterogeneity of tumors at high resolution. However, in addition to this, there is asynchrony in the development process of tumor cells in organisms—that is, the cycle states of different cells are not the same [4]. Bulk-based sequencing is performed on the whole tissue, in which cells with different cycle states are contained, thus also masking temporal heterogeneity between cells. Hence, at present, a large number of methods have emerged for single-cell trajectory inference, which can infer the position of each cell on the pseudo-time axis through algorithms based on expression profiles, so as to reproduce the trajectory of cell differentiation over time. The traditional clinical stage can reflect the time status of the development of a tumor; therefore, single-cell pseudo-time trajectory inference combined with clinical stages can more clearly analyze the dynamic process of tumor development, so as to more accurately discover the biological factors that promote tumor evolution and, finally, to promote the development of clinical treatment.

Therefore, our study emphasized the dynamic development process of OSCC. Unsupervised clustering based on single-cell transcriptomics was first performed to analyze the heterogeneity of OSCC. Pseudo-time trajectory inference was subsequently performed based on the unsupervised clustering results to dissect the complex development trajectory of OSCC from the early to the late stages. Next, to validate the cell trajectory, an analysis of receptor–ligand-based cell communication was conducted. At present, the single-cell field is under the transition from descriptive biology to predictive biology [5]; therefore, in order to promote the theory in clinical applications, we used bulk RNA-Seq data from the TCGA HNSC cohort to analyze the effects of different cell cluster compositions on patients’ survival time. More importantly, we proposed pseudo-time score that can combine heterogeneity with the pseudo-time occupied by cell clusters and can quantify the status of patients during cancer development. We also used the external cohort from the ICGC [6] database to validate the pseudo-time score. Finally, based on the heterogeneity and complex development trajectory of OSCC, we discovered potential targeted therapeutic drugs that can not only personalize the treatment according to the individual’s cell cluster composition but also block the development and deterioration of OSCC as much as possible in order to jointly improve the current situation of clinical treatment of advanced OSCC.

## 2. Results

### 2.1. High Heterogeneity of OSCC

**Figure 1:**
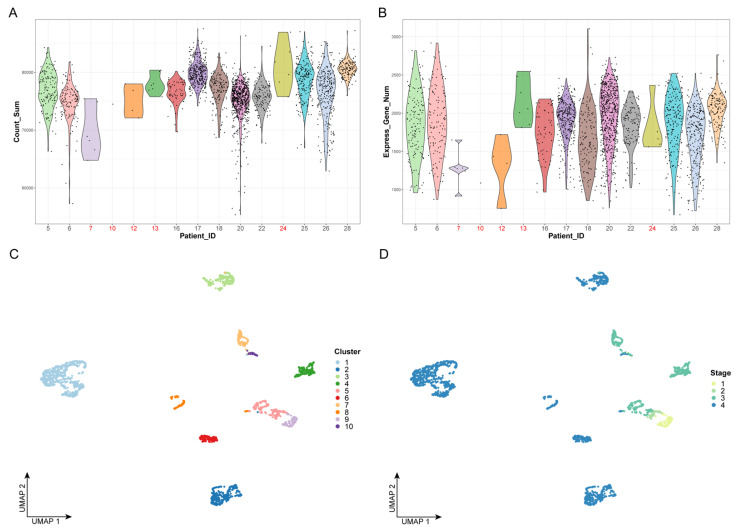
Data preprocessing and unsupervised clustering: (**A**) Violin plot of gene expression counts. The *x*-axis represents the patient ID, while the *y*-axis represents the sum values of gene expression counts. The dots represent the cells. Patient IDs that failed quality control are marked in red. (**B**) Violin plot of expressed gene numbers. The *x*-axis represents the patient ID, while the *y*-axis represents the number of expressed genes. The dots represent the cells. Patient IDs that failed quality control are marked in red. (**C**,**D**) The results of cell clustering. The dots represent the cells. The *x*- and *y*-axes represent the two dimensionalities of UMAP, respectively. Cells are colored by cluster label in panel C and by clinical stage in panel D.

We first performed quality control on the data by examining the number of cells and the gene expression levels of all patients; we removed five patients, including 24 cells, which are represented in red in Figure 1A,B. Next, we extracted 12,000 highly variable genes from a total of 23,686 genes to perform feature selection and to improve the accuracy of downstream analysis. Principal component analysis (PCA), which is a widely used dimensionality-reduction method, was used to process the expression profiles of highly variant genes. The optimal number of dimensions was determined by the elbow method, as shown in Figure 1C. Finally, the top 20 principal components were selected for unsupervised clustering (Figure S1).

Based on the top 20 principal components, we performed unsupervised clustering on 2176 cancer cells, which were divided into 10 clusters (Figure 1C,D). We found that, with the progression of the clinical stages, the number of cell clusters also gradually increased, indicating that there is greater heterogeneity in advanced OSCC (Figure 1D). This could explain the difficulty of completely curing advanced OSCC with current clinical treatments.

### 2.2. Different Development Fates of OSCC Cells

**Figure 2:**
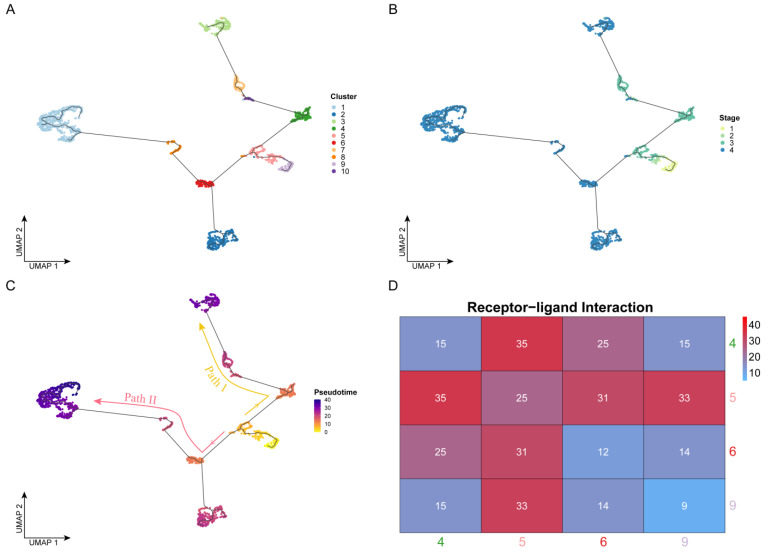
Cell trajectory inference: (**A**–**C**) Cell development trajectory. The dots represent the cells. The *x*- and *y*-axes represent the two dimensionalities of UMAP, respectively. Cells are colored by cluster label in panel A, by clinical stage in panel B, and by pseudo-time value in panel C. Based on the pseudo-time, the cell evolution direction could be determined, which was mainly divided into two paths: Path I (yellow) and Path II (red). (**D**) Results of cell communication analysis. The axis represents the four cell clusters near the branch point. The color and the number labeled by this heatmap were determined by the receptor–ligand pair number between two cell clusters.

To reveal the developmental process of OSCC from the early to the advanced stages, we performed cell trajectory inference analysis based on pseudo-time for all cells using Monocle (Figure 2A,C). It is worth noting that the development of OSCC cells did not follow a single trajectory and was divided into two paths by a branch point. In terms of cell clusters (Figure 2A), clusters 9 and 5 appeared before the branch point, Path I developed along with the order of clusters 4, 10, 7, and 3, and Path II developed along with the order of clusters 6 (2), 8, and 1. From the perspective of clinical stages (Figure 2B), the temporal order was followed by stages I, II, III, and IV, consistent with the actual clinical development progress. Cells from stages I and II mainly appeared before the branch point, which appeared at stage III. Cells developed along the two paths and eventually deteriorated into stage IV. However, the two paths had different deterioration trends. Some cells from the branch point directly developed into stage IV (Path II), while the other cells remained in stage III (Path I) for a long time before eventually developing to stage IV. Therefore, identifying the key genes driving these two paths, with the goal of finding appropriate drugs to prevent the progression of cancer, would make significant progress in improving the current treatment of advanced OSCC.

### 2.3. Characteristic Analysis of Intercellular Communication

In the whole body or a specific tissue, the complex interactive relationships between cells constitute the cell communication network, which can reflect the tightness of the connection between different cells. Based on this, we hypothesized that the stronger the intercellular communication, the closer the connection, and the closer it is in the time series.

Therefore, to verify the accuracy of the cell trajectory inference, we performed receptor–ligand-based cell communication analysis. Throughout the trajectory, directly connected cells should tend to interact more tightly, while indirectly connected cells should have a weaker interaction because they require the transition of the intermediate cells—that is, there should be a tighter interaction between adjacent cell clusters in the trajectory. Here, we used CellPhoneDB to perform receptor–ligand-based analysis on cells of four clusters (4, 5, 6, and 9) near the branch point that separates the two paths in order to reveal their intercellular interaction relationships. The level of intercellular interactions was measured by the number of receptor–ligand pairs. As shown in Figure 2D, from the overall point of view, the tightness of the connection between cell clusters 5-4, 5-6, and 5-9 was much higher than that between the other cluster pairs, and as expected, these cell clusters were located adjacent to one another in the pseudo-time trajectory. In addition, we found that the interaction strength between clusters 4 and 5 was the highest. These two cell clusters not only were located close to one another in the pseudo-time trajectory but also belonged to the same stage (III), so the intercellular communication connection between them should also have been closed. The number of receptor–ligand pairs between clusters 5 and 9 was the second highest, at 33. These cells were from stages I, II, and III, but they were all located before the branch point, so the level of communication between them was higher than that of others after the branch point.

In conclusion, the analysis of intercellular communication based on receptor–ligand pairs was consistent with the results of cell trajectory based on pseudo-time and, thus, could further validate the extremely complex landscape during the development of OSCC cells from the early to the late stages.

### 2.4. Biological Factors Driving the Two Paths at the Branch Point in the Cell Development Trajectory

**Figure 3:**
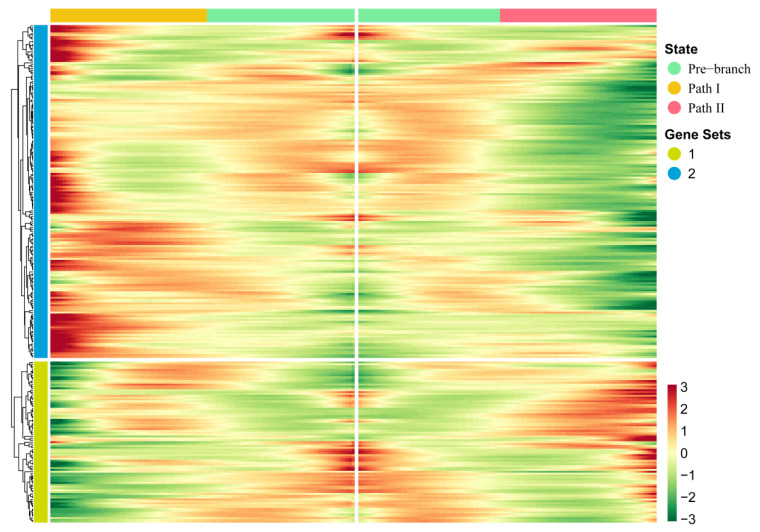
Key genes driving the occurrence of the branch point: The heatmap of gene expression levels during the progression of OSCC. Gene clusters were generated by hierarchical clustering.

In order to further explore the driving factors of the branch point that separated Paths I and II, we used the BEAM method to perform pseudo-time-based differential gene expression analysis on four cell clusters (4, 5, 6, and 9) near the branch point. As a result, we identified 267 genes that showed significant fluctuation in their expression at the branch point (Figure 3; Table S2). Compared with traditional differential gene expression analysis, BEAM combines pseudo-time to reflect the continuous changes in gene expression. Through hierarchical clustering of these genes, all genes were divided into two gene sets. We found that the majority of these genes showed mutually exclusive expression characteristics in two directions—that is, high expression characteristics in Path I but low expression characteristics in Path II, or vice versa—indicating that these genes regulate two mutually exclusive cell fates. In addition to this, genes in a gene set of hierarchical clustering usually have co-expression characteristics that may co-regulate some biological functions.

**Figure 4:**
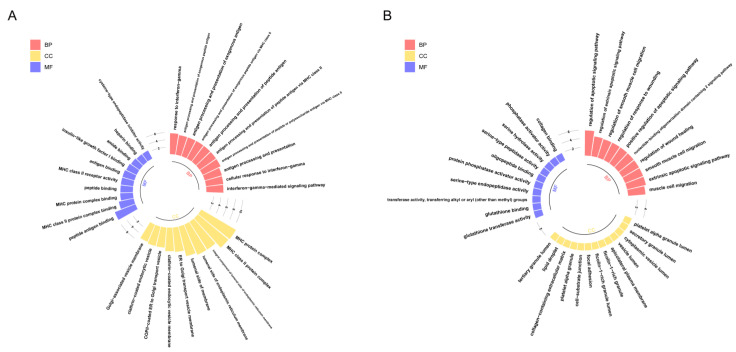
GO enrichment analysis of key genes: The results of GO enrichment analysis of gene clusters 1 and 2 are shown in panels (**A**,**B**), respectively. The *x*-axis represents GO terms, which are displayed in three colors according to the three types (BPs, CCs, and MFs), while the *y*-axis represents -log_10_(p.adjust). Only the 10 most significant GO terms of each type are shown here.

Therefore, in order to reveal the biological functions regulated by these genes, we performed Gene Ontology (GO) enrichment analysis on two gene sets (Figure 4). The results showed that genes highly expressed in Path I (gene cluster 2) were significantly enriched mainly in biological processes, such as the cell migration and apoptosis signal regulation pathways (Figure 4B). The important role of cell migration and apoptosis in the development and progression of OSCC has been confirmed [1]. Marker genes regulating the cell cycle, apoptosis, and migration have differential expression in OSCC patients or are significantly associated with prognosis [1], such as survivin and heat shock proteins (HSPs) associated with apoptosis. Survivin is a member of the inhibitor of apoptosis proteins (IAP) family that inhibits capase 3, 7, and 9, and its expression is higher in OSCC patients than in epithelial dysplasia patients. High expression of heat shock protein 27 (HSP27) is associated with a better prognosis. The expression of urokinase plasminogen activator receptor (UPAR)—a marker gene associated with cell migration—was negatively correlated with prognosis [1]. Therefore, the genes highly expressed in Path I suggest that OSCC cells may have active metastatic characteristics in clinical stage III. The genes highly expressed in Path II (gene cluster 1) were mainly enriched in biological functions related to MHC class II in BPs (biological processes), CCs (cellular components), and MFs (molecular functions) (Figure 4A). MHC is a collective term for a group of genes encoding major histocompatibility antigens in animals, also known as HLA in humans, which is also involved in the immune process of the body as an antigen. There is literature confirming the upregulation of class II molecules of the major histocompatibility complex (MHC) by keratinocytes in oral squamous cell carcinoma [7]. However, the significance of its high expression in advanced OSCC is currently unclear. Another study confirmed that the keratinocyte line expressing MHC II has the characteristics of the absence of CD80 and CD86 in head and neck cancer, which may be a way for tumors to evade immune surveillance [8].

### 2.5. Relationship between Prognosis and Heterogeneity of Advanced OSCC

Our findings show that advanced OSCC has more significant heterogeneity than early OSCC, and it is difficult to characterize this heterogeneity via traditional bulk research. Therefore, to further dissect the relationship between this heterogeneity and prognosis, we integrated clinical information and expression profiles from the TCGA database to explore the prognosis of stage III and IV patients with greater heterogeneity, which would also indirectly verify the accuracy of our predicted cell clusters.

**Figure 5:**
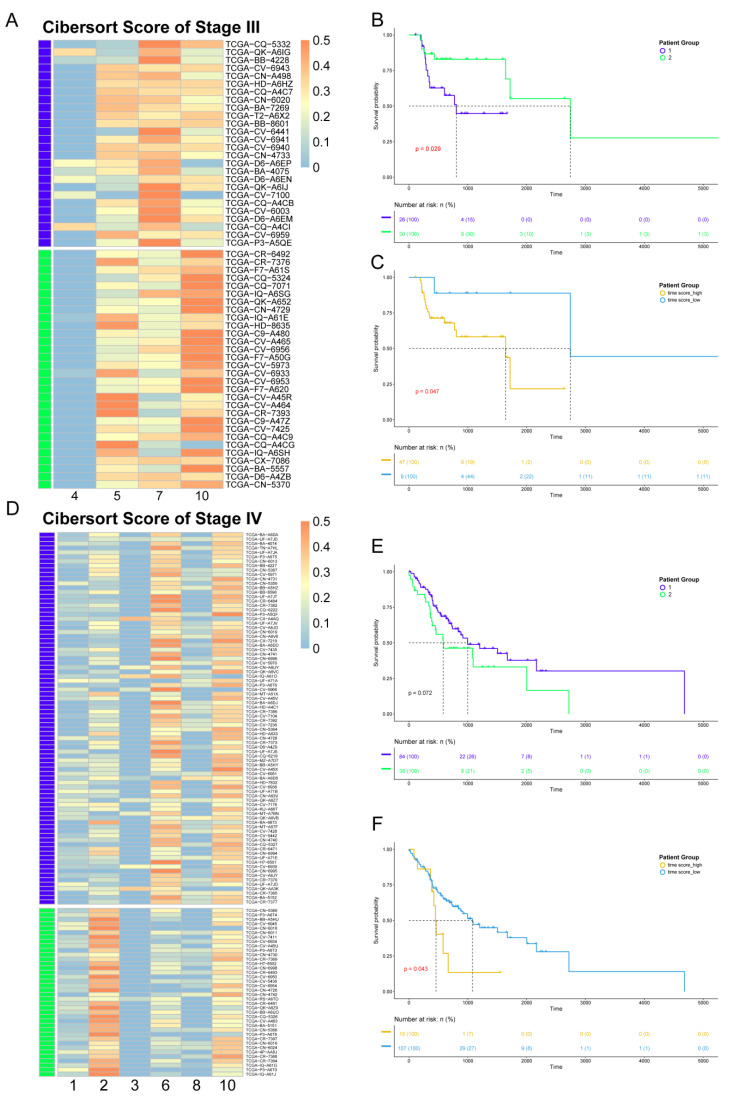
Association between heterogeneity and clinical prognosis. (**A**,**D**) The results of CIBERSORT. Patients were divided into two groups by hierarchical clustering. Patients in stage III are shown in panel A, while patients in stage IV are shown in panel D. (**B**,**E**) Survival analysis. The survival curve is shown above, with the *x*-axis representing the survival time (days), the *y*-axis representing the survival probability, and the censored values indicated by “+” in the curve. The color of the curve is consistent with panel A (**D**) and represents two patient groups. Below is the risk table showing the number (or percentage) of survivors at each time point. The results of patients in stage III are shown in panel B, while those of patients in stage IV are shown in panel E. (**C**,**F**) Survival analysis based on pseudo-time score for grouping. The color of the curve represents the two patient groups, which are distinguished by their pseudo-time score. The result of patients in stage III is shown in panel C, while those of patients in stage IV are shown in panel F.

Since the data from the TCGA database are at the bulk level, we first mapped corresponding cell clusters to bulk expression profiles to infer the cell cluster composition of each patient. Based on the custom background gene sets (Table S3) derived from differential expression analysis, CIBERSORT was performed for stage III and IV patients from TCGA (Table S4). Next, in order to reveal the impact of cell cluster composition on the prognosis of patients, we performed unsupervised hierarchical clustering based on the results of CIBERSORT. The patients in stages III and IV were divided into two groups. Each patient group had similar cell cluster composition, and there were significant differences between the patient groups (Figure 5A,D). For patients in stage III, including four clusters, group 1 mainly contained cell cluster 7, while group 2 mainly contained cell clusters 5 and 10. For patients in stage IV, composed of six clusters, group 1 mainly contained cell cluster 6, while group 2 mainly contained cell cluster 2.

Survival analysis was performed for the two groups of patients in stages III and IV. The results showed differences in survival time between the two groups (Figure 5B,E), indicating that different cell compositions could impact prognosis. For patients in stage III, the survival time of patient group 1 was shorter, while for stage IV, the survival time of patient group 1 was longer.

In order to explore the reason(s) that this heterogeneity has an impact on the survival time of patients, we constructed pseudo-time score by combining cell trajectory inference to quantify the temporal status of each patient group. The pseudo-time score considers both the cell composition of patients and the development order of each cell cluster in the same clinical stage. The higher the score, the closer the patient is to the advanced stage. The results showed that, in stage III, S_patient group 1_ = 16.35 and S_patient group 2_ = 15.19. As for stage IV, S_patient group 1_ = 18.59 and S_patient group 2_ = 20.44. According to the survival analysis, the groups with smaller pseudo-time score tended to have a better prognosis. Therefore, the difference in prognosis status between patient groups lies in the different cell compositions, which contribute to the different temporal status during the development of cancer. Our results also show that even patients at the same clinical stage would have many differences in cell composition and prognosis.

To further analyze the relationship between the pseudo-time score and patients’ prognosis, we regrouped patients according to their pseudo-time score, and the results showed that the pseudo-time score could be used as the marker to distinguish survival time in both stage III and stage IV patients, with *p*-values of 0.047 and 0.043, respectively, as measured by the log-rank test. The patient groups with the higher score had the worse prognosis (Figure 5C,F).

To confirm the above results, the same methods were used to process the dataset from ICGC, and the cell cluster composition inference of patients was performed using CIBERSORT based on the same background gene sets. Then, these patients were divided into two groups based on their pseudo-time score, and survival analysis also illustrated that the two groups of patients showed significant differences in survival time (*p* = 0.049, log-rank test) (Figure S2).

In summary, the pseudo-time score that we constructed has a significant correlation with prognosis, can be used as a prognostic marker, and is robust across multiple datasets.

### 2.6. Identification of Candidate Drugs Based on PPI Networks

Transcriptomic analysis at the single-cell level with high resolution is a way to improve the efficacy of medication by dissecting the diverse cell clusters in the tumor and by selecting the targeted therapy strategy. In addition, it is also essential to find key genes with synchronous expression changes during the development of OSCC and to use these genes as potential targets to discover blockers to slow or even block the deterioration of OSCC in order to prolong the treatment time in clinical practice and to maximize the lifespan of patients. Therefore, we performed drug discovery in the following two respects: (1) searching for drugs to block OSCC progression and (2) searching for cell-cluster-specific drugs to achieve targeted therapy.

**Figure 6:**
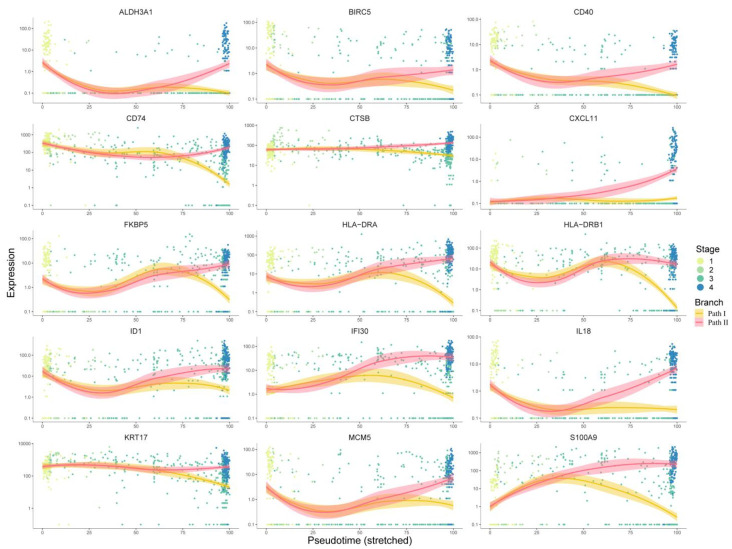
Analysis of 15 target genes’ expression trends: This figure shows the expression trends of genes progressing with pseudo-time. The *x*-axis represents pseudo-time, while the *y*-axis represents the gene expression level. Dots represent cells, which are colored according to clinical stages. The curves were fitted by gene expression level, and the colors represent two different development paths in the cell trajectory.

For the first respect, 267 key genes at the branch point revealed by the BEAM analysis were first used for protein–protein interaction (PPI) network construction using the STRING [9] database. Cytoscape software was used for network visualization (Figure S3). There were 218 nodes and 649 edges in the PPI network. We used the cytoHubba [10] plugin built in Cytoscape to identify the hub genes, and all 12 topological analysis methods were taken into account to improve the robustness. Then, the expression trends of these hub genes with pseudo-time were manually checked, and only genes showing a significant trend of upregulation in Path II and downregulation in Path I were retained as marker hub genes. As a result, 15 marker hub genes were identified (Figure 6). Except for ALDH3A1, CD40, CXCL11, HLA-DRA, and HLA-DRB have been validated by the literature to have positive relationships between expression and prognosis, so we removed them from candidate drug targets, while the others presented malignant characteristics (Table S5). Finally, using the 10 remaining genes as targets, 195 drugs were extracted from integrated drug–target relationships, including first-line antitumor drugs, such as cisplatin, fluorouracil, methotrexate, gemcitabine, p-phenylenediamine, etc. (Table S6, Sheet 1). Among them, 90 drugs were validated based on CCLE experimental data (Table S6, Sheet 2), and 77 of the remaining 105 drugs were validated based on the literature (Table S6, Sheets 2 and 3). The drugs targeting multiple targets were paid more attention to. Cyclosporine and valproic acid (VPA) targeted all 10 proteins. In recent years, VPA has been found to be a histone deacetylase inhibitor (HDACi). Many experimental studies have shown that VPA can inhibit the growth and proliferation of tumor cells by inducing cell cycle arrest, apoptosis, and differentiation and by inhibiting tumor angiogenesis and metastasis [11,12]. It is known that the combination of cisplatin (CDDP) and cetuximab (CX) is one of the standard first-line treatments for OSCC. However, this therapeutic regimen is often associated with resistance, suggesting that new combinatorial strategies need to be improved. Federica Iannelli et al. demonstrated that the introduction of VPA to the conventional treatment for recurrent/metastatic HNSCC represents an innovative and feasible antitumor strategy that warrants further clinical evaluation [13]. Another study showed VPA acting as a histone deacetylase inhibitor (HDI) in OSCC cells and normal human keratinocytes (HKs), potentiating the cytotoxic effect of cisplatin in OSCC cell lines and decreasing the viability of OSCC cells as compared to HKs [14]. Taken together, these results provide initial evidence that VPA might be a valuable drug in the development of better therapeutic regimens for HNSCC.

**Figure 7:**
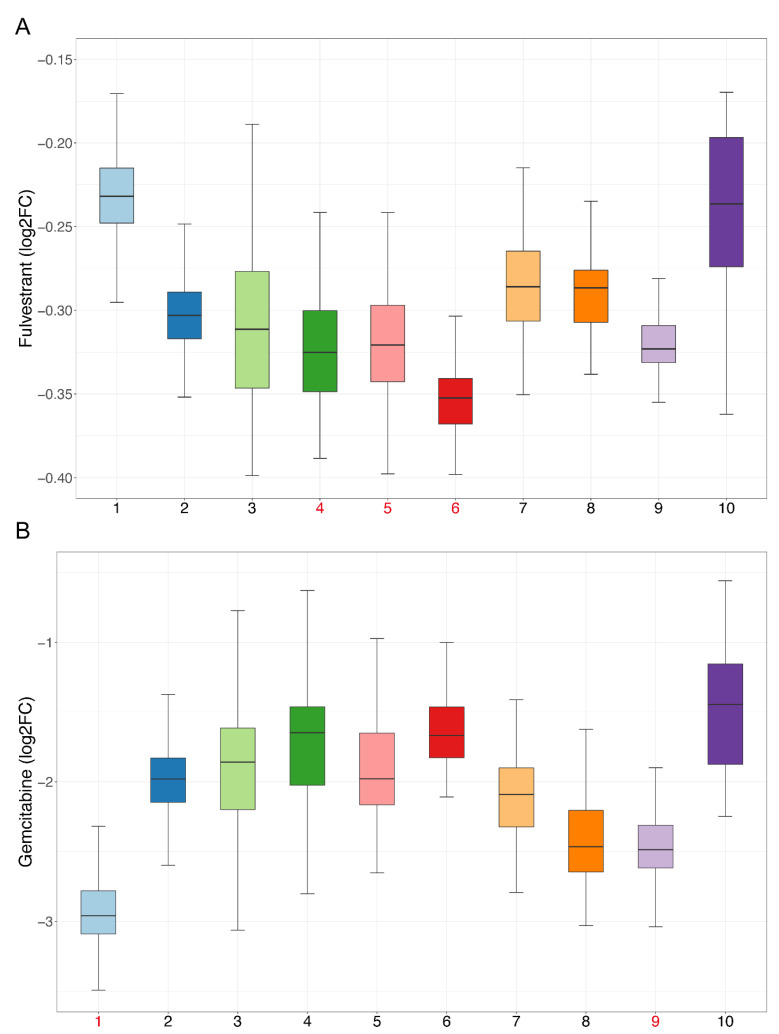
Sensitivity of candidate drugs: The *x*-axis represents cell clusters, and the *y*-axis represents the logFC (drug sensitivity data from the CCLE are represented using relative log fold-change values of cell lines’ viability to DMSO). Cell clusters with the highest drug sensitivity mentioned in our study are marked in red. (**A**) Sensitivity of fulvestrant. (**B**) Sensitivity of gemcitabine.

We performed drug sensitivity predictions at single-cell resolution for these drugs, and the drug sensitivity of cell clusters was represented by the mean value of drug sensitivity of the cells in each cluster. According to the pseudo-time trajectory, all cell clusters were divided at the branch point into Paths I and II. Fifty-three drugs were predicted to have higher sensitivity in Path II, suggesting that these drugs are more effective at inhibiting malignant developmental processes (Table S6, Sheet 4). Of these, we found that fulvestrant simultaneously exhibited the highest drug sensitivity in cell clusters 4, 5, and 6, which appeared near the branch point and, thus, likely represented an earlier exacerbation progression (Figure 7A). A study has shown that estrogen can participate in the progression of precancerous lesions of HNSC by inhibiting apoptosis and by promoting the proliferation of advanced HNSC cells [15]. Antiestrogen may be beneficial as a chemopreventive agent for HNSC [15], and fulvestrant is an antiestrogen drug, so our results were consistent with those of the previous study.

For the second respect, marker genes were first calculated using the SC3 [16] method for all cell clusters. With manual examination, a total of 459 genes remained after statistical filtering (Table S7, Sheet 1). These genes showed significant upregulation in specific cell clusters, so they could be used as essential cell-cluster-specific marker genes for drug discovery. As described above, the PPI networks of each cell cluster were constructed (Figure S4), and then, 12 topological methods from cytoHubba were taken into account for identification of the hub genes. Then, 76 hub genes were filtered (Table S7, Sheet 2), and 478 drugs were ultimately discovered (Table S8). Some of these drugs overlap with the blocking drugs found in our study and are all first-line anticancer drugs, such as valproic acid, cisplatin, fluorouracil, methotrexate, temozolomide, etc. Based on CCLE experimental data, 195 drugs were validated as being effective in HNSC cell lines (Table S9, Sheet 1), and 168 of the remaining 283 drugs were validated through the literature (Table S9, Sheets 1 and 2). Many previous studies have shown that the combined use of certain drugs can enhance their effects; for example, the combination of curcumin and copper can enhance the inhibitory effect on the migration and activity of OSCC cells [17]. Our findings show that curcumin and copper can be used for blocking the development of OSCC and targeting cell cluster 1, indicating that the two have the potential for combination, and are consistent with the results of the previous study. Next, we analyzed the sensitivity of drugs and extracted drugs that had higher sensitivity in their targeting of cell clusters. Finally, there were 102 drugs, including many drugs that have been proven to be effective for HNSC treatment (Table S9, Sheet 3). Cell cluster 1 is at the end of the pseudo-time trajectory and, thus, represents highly advanced OSCC cells. Among the selected drugs, there were 71 drugs targeting cell cluster 1, including common anticancer drugs, such as paclitaxel, gemcitabine, carboplatin, decitabine, etc. (Figure 7B). We performed literature validation for all of these drugs, and 91 of the 102 had been reported in previous studies for cancer treatment or combined medication. Therefore, the candidate drugs discovered in our study could be of great significance to changing the current situation of treatment for advanced OSCC.

## 3. Methods

### 3.1. Data and Preprocessing

The single-cell RNA sequencing data of OSCC used in this study were obtained from the GEO database (GSE103322), with a total of 5902 cells from 15 patients [18] including all clinical stages. These data were preprocessed using the method described in the article published by Sidhart V. Puram et al. [18], and all cells were accurately classified into cancer and non-cancer types. The gene expression did not conform to a normal distribution in five patients due to the low number of cells, which may have had a bad impact on the accuracy of the downstream analysis; therefore, these five patients were removed. There were 2200 cancer cells in total. The detailed information of all patients is shown in Table S1. Because our study focused on cancer cells, non-cancer cells were removed.

Bulk sequencing data were obtained from the HNSC cohort of the TCGA database and contained a total of 501 patients.

The dataset used for pseudo-time score validation was obtained from the ICGC database (ORCA-IN, Sequence-Based Gene Expression).

### 3.2. Unsupervised Clustering of Cells

We used the R package Seurat v4.0 [19]—a toolkit developed specifically for single-cell data. First, the R function “VlnPlot” was used to evaluate the gene expression levels of all cells to ensure that the gene expression level was distributed in an approximately Gaussian manner in each patient, so as to reduce the impact of individual differences on downstream analysis. Then, the expression profiles were log-normalized using the R function “NormalizeData”, and 12,000 high variant genes were identified from 23,686 genes using the R function “FindVariableFeatures”. Genes with high variation can better reflect the biological similarities and differences between cells, which is conducive to improving the accuracy of downstream unsupervised clustering. Next, we used the R function “ScaleData” to scale the expression of highly variant genes in order to balance the weight of genes in the downstream analysis. Because single-cell data are usually sparser compared with bulk sequencing and their redundancy is higher, principal component analysis (PCA) is an important and essential step in single-cell transcriptome analysis. PCA can effectively remove data noise, extract important information, and improve the accuracy and speed of downstream analysis. PCA was performed using the R function “RunPCA” on the expression profiles of highly variable genes, and then, the R function “ElbowPlot”—which ranks the principal components based on the percentage of variance explained by each component—was used to determine the optimal dimension number of the data. The optimal number of principal components would appear near the “elbow” (i.e., the inflection point). Unsupervised clustering based on the top 20 principal components was implemented for all cells using the R function “FindClusters”.

### 3.3. Trajectory Inference of Cell Development

We used the R package Monocle [20]—a powerful tool for single-cell RNA-Seq data processing and cell trajectory inference—for analysis. Monocle uses algorithms to learn the gene expression changes that each cell experiences during a state transition, to mine the overall trajectory, and then to place each cell at the appropriate location in the trajectory. In addition, Monocle can combine with UMAP (Uniform Manifold Approximation and Projection) [21] so as to make the cell trajectory more intuitive. Therefore, all cells were first embedded by UMAP based on unsupervised clustering results.

The R functions “learn_graph” and “order_cells” were used for the inference of cell trajectory, both of which used default parameter settings. Subsequently, the R function “plot_cells” was used to visualize the results of cell trajectory inference with the pseudo-time.

### 3.4. Gene Expression Analysis at the Branch Point of the Trajectory

The BEAM (branch expression analysis modeling) [22] algorithm provided by Monocle can analyze the pseudo-time-based gene expression changes at the branch point of the trajectory, revealing the important genes that drive the occurrence of cell trajectory division. The threshold was set as *q*-value < 1 × 10^−8^. This stricter threshold was designed to identify essential genes more accurately. In addition, only the genes expressed in more than 20% of cells were retained in order to screen widely expressed genes in cells.

In order to reveal the biological functions of these genes, they were first divided into two gene sets by hierarchical clustering. The genes in the same gene set would have co-expression characteristics and may regulate similar biological processes. Thus, Gene Ontology (GO) [23] enrichment analysis was performed for each gene set separately using the R package clusterProfiler [24] with the threshold *p*.adjust < 0.05 and *q*-value < 0.05.

### 3.5. Cell Communication Analysis

In order to verify the results of cell trajectory inference and to further determine our conclusions, receptor–ligand-based cell communication analysis was performed using the Python package CellPhoneDB [25] for cell clusters adjacent to the branch point. The iteration parameter was set to 2000. The number of receptor–ligand interaction pairs was visualized using a heatmap, and a dot plot was used to show pairs with statistical *p*-values < 0.05 and mean expression > 0.

### 3.6. Survival Analysis

In order to reveal the impact of heterogeneity on the survival status of patients, we downloaded the RNA-Seq data from the HNSC cohort with clinical information of patients from the TCGA [26] database. CIBERSORT [27] is a linear support-vector regression-based deconvolution algorithm that enables researchers to perform sample annotation based on a set of background genes. Hence, the selection of the background gene set would have a great impact on the accuracy of the downstream analysis. Since the type of expression profile derived from the TCGA database is at the bulk level, we used the differentially expressed genes that can represent the characteristics of each cluster separately as a background to infer the proportion of cell clusters of each bulk sample using the R package CIBERSORT. For stage III, the threshold for gene background screening was *p*-value < 0.01 and log_2_fc > 1.5. Since patients in stage IV have greater heterogeneity, the threshold was set as *p*-value < 1 × 10^−4^ and log_2_fc > 1.5. This stricter threshold can help identify the genes that represent the characteristics of each cell cluster more effectively and can improve the accuracy of the proportion inference. The results of CIBERSORT were subsequently filtered by setting the threshold as *p* < 0.05 and correlation > 0.3. The Ward.D algorithm [28] was used to perform hierarchical clustering to divide patients in stage III and stage IV into two groups. The survival [29] and survminer [30] R packages were used for survival analysis, plotting of the Kaplan–Meier curve, and the log-rank statistical test.

### 3.7. Pseudo-Time Score

Here, we hypothesized that patients in the same stage would also have relatively early or advanced cancer cells due to the temporal heterogeneity and that cancer cells in the advanced stage would have more malignant features, leading to a worse prognosis. Therefore, in order to prove our hypothesis, based on the previous trajectory inference results, the pseudo-time score was constructed to quantify the temporal status of each patient. The formula was S = ∑P_i_ × T_i_, where P_i_ denotes the proportion of cell cluster i of the patient, while T_i_ denotes the pseudo-time value of cell cluster i. P_i_ was calculated using CIBERSORT. T_i_ is the average pseudo-time value of each cell from the cell cluster i, which can be calculated by Monocle. Specifically, for patients in stage III, S = ∑P_i_ × T_i_ (i = 4, 5, 7, or 10), while for patients in stage IV, S = ∑P_i_ × T_i_ (i = 1, 2, 3, 6, 8, or 10). As for the patient groups, the pseudo-time score is the average value of S of each patient from each group.

In order to further reveal the relationship between pseudo-time score and prognosis, all patients were automatically grouped based on their pseudo-time score using survminer to perform survival analysis. Similarly, statistical significance was tested using the log-rank method.

The dataset from the ICGC database was used for further validation. This dataset contains 40 patients, including a stage II patient, 3 stage III patients, and 36 stage IV patients. The cell cluster composition of each patient was inferred using CIBERSORT, based on the same background gene sets as the TCGA data above, and then, the pseudo-time score of each patient was calculated and the patients were grouped for survival analysis as described above.

### 3.8. Drug Discovery

There may often be many cell clusters in a patient under the single-cell resolution. For such a complex system of multi-cell clusters, we should discover specific drugs for each cell cluster, so as to select multitarget drugs or drug combinations according to the heterogeneity of patients for the elimination of all cell clusters of patients—rather than just the dominant cell cluster, which can often cause drug resistance. Therefore, we first screened specific drugs for each cell cluster. Secondly, discovering drugs to block or delay the development of OSCC would also be an idea to effectively improve the cure rate.

The discovery of drug targets is the first step. The marker genes of each cell cluster were identified using the R package SC3 [16], with the threshold set as *p*-value < 0.01 and AUROC (the area under the receiver operating characteristic) >0.8. Then, all marker genes were manually checked to ensure that they had significantly high expression characteristics in specific cell clusters. In biomolecular networks, hub nodes often have crucial biological significance, so we used hub genes in the network as targets to find candidate drugs. First, we used STRING [9] and selected the default threshold to construct protein–protein interaction (PPI) networks based on the key genes that drive the occurrence of trajectory branching from BEAM analysis and cell-cluster-specific marker genes from SC3. The visualization of PPI networks and further topological analysis were based on Cytoscape [31] software. To identify hub genes more accurately, we deeply mined the PPI networks based on 12 topological analysis methods built into the cytoHubba [10] plugin. The hub genes were defined as the intersection genes of the top 50% of each of the 12 analysis methods and were used as targets for drug discovery.

Here, we collected 216,428 drug–target relationships including 21,650 human targets and 2470 approved drugs from seven commonly used data sources: the DrugBank database (v5.1.9) [32], the Therapeutic Target Database (TTD) [33], the BindingDB database [34], the PharmGKB database [35], the Drug–Gene Interaction Database (DGIdb, v4.2) [36], the IUPHAR/BPS Guide to PHARMACOLOGY [37], and the Comparative Toxicogenomics Database (CTD) [38]. Finally, using the aforementioned genes as targets, we obtained the preliminary candidate drugs.

### 3.9. Validation of Candidate Drugs

To validate the candidate drugs identified in our study, we combined experimental data, computational methods, and previous literature to assess the effectiveness of the candidate drugs.

The drug sensitivity experimental data and cell line expression profiles were first obtained from the Cancer Cell Line Encyclopedia (CCLE) [39] database, and all OSCC cell lines were extracted. In order to validate drug effectiveness from experiments indirectly, we computed the mean drug sensitivity of all OSCC cell lines for each drug contained in the CCLE. For drugs that were not included or showed no efficacy in the CCLE, additional literature validation was performed.

In order to reveal the drug specificity during the development of OSCC and of cell clusters, we used the R package oncoPredict [40]—a powerful tool for predicting drug responses based on background data (here, we used drug sensitivity and expression data from the CCLE)—to calculate the drug sensitivity of each cell. Then, the drug sensitivity of each cell cluster was defined as the mean drug sensitivity of cells from this cell cluster. We extracted blocking drugs and cell-specific drugs with high specificity. Drug sensitivity data from the CCLE were represented using the relative log fold-change (logFC) values of cell lines’ viability to DMSO. Therefore, for blocking drugs, the following conditions were used for screening: (1) The mean logFC in all cells was lower than 0. (2) The mean logFC of cell clusters located in Path II was lower than that in Path I. For cell-cluster-specific drugs, we screened by the following criteria: (1) The mean logFC in all cells was lower than 0. (2) The mean logFC of targeting cell clusters was lower than that of non-targeting cell clusters.

## 4. Discussion

The effective curing of advanced OSCC has been a clinical challenge because of its high heterogeneity and metastatic characteristics. Current precision treatment strategies at the single-cell level only focus on static heterogeneity and do not consider dynamic characteristics due to tumor cells’ development and evolution. Therefore, our study applied traditional single-cell transcriptome analysis and dynamic cell trajectory inference theory to further explore heterogeneity and precise treatment strategies for OSCC.

During the cell clustering analysis, we found that, with the development of tumor cells, their heterogeneity became greater and greater. This complex heterogeneity reflects the characteristics of multiple evolutionary modes of tumor cells in the same stage that have different sensitivities to chemotherapeutic drugs, which is consistent with the fact that advanced OSCC is difficult to cure. Therefore, we innovatively quantified the drug sensitivity of specific cell clusters and selected drugs with high sensitivities in those specific cell clusters.

The cell trajectory inference with pseudo-time reflects the temporal heterogeneity of OSCC, which forms a complex exacerbation process composed of two paths through a branch point and gives the temporal characteristics of each cell. We integrated this temporal characteristic and cell cluster composition of patients to establish the concept of patients’ time score, with significant implications for prognosis—that is, the lower the time score, the better the prognosis. Based on this, it can be seen that even patients in the same clinical stage have different temporal status.

In addition, the patient’s response to drug treatment is also dynamic, so RNA-Seq can be performed at different time points during the treatment to focus on the disease progression based on our proposed pipeline. If the time score decreases gradually with the treatment, the prognosis of the patients will be better; otherwise, the medication strategy needs to be adjusted.

Finally, based on the key genes driving the differentiation of cell development trajectory and the cell-cluster-specific marker genes, we used biomolecular network theory and topological analysis to mine hub genes with extremely important biological significance, which were used as targets to find candidate drugs. As a result, there were a total of 167 drugs targeting key genes at the branch point in cell trajectory, which could be used to delay or block the further progression of OSCC. There were a total of 363 cell-cluster-specific drugs, which could be used for targeting medication based on patients’ cell composition. These drugs can be combined to treat patients with multiple cell clusters, but some drugs (such as artenimol) can also target multiple cell clusters at the same time. We prefer the latter, so as to maximize the efficiency of the medication while reducing the side effects. Candidate drugs were validated by both literature and computational methods combined with experimental data.

In conclusion, our research provides a new pipeline to dissect the complex heterogeneity of cancer from a dynamic point of view. More importantly, our study proposes the concept of patients’ pseudo-time score, which has great clinical value. A series of precision medicine insights for the clinical treatment practice of OSCC were presented—that is, the selection of appropriate drugs for tumor control, which aims to not only block or delay the progression of OSCC but also comprehensively kill various cancer cells. This strategy may also be adapted for other cancers in the future.

However, due to data limitations, no more in-depth research has been carried out. In addition, due to the absence of clinical trials, this study has some limitations. As single-cell spatial sequencing and cell trajectory inference technologies mature, we have reason to believe that the challenges in completely curing OSCC or other cancers will eventually be overcome.

## 5. Conclusions

Our study confirms the complex heterogeneity of OSCC both statically and dynamically. Advanced-stage patients have greater heterogeneity than early stage patients. The development of OSCC is not a simple pathway, and cell trajectory inference confirms its complex dynamic landscape, which contains multiple developmental pathways. According to this, the proposed pseudo-time score is closely related to the patient’s prognosis and has good prognostic prediction potential, as validated by external datasets. We searched for candidate drugs for the treatment and blocking of OSCC, most of which were validated in the literature and via computational methods based on experimental data. In conclusion, our study comprehensively parses the developmental characteristics of OSCC, offering new insights into the basic research and clinical treatment of OSCC.

## Data Availability

The expression data are available through the Gene Expression Omnibus with accession number GSE103322.

## Supplementary Materials

The following supporting information can be downloaded at: https://www.mdpi.com/article/10.3390/cancers14194801/s1, Figure S1. Principle component selection. The x-axis represents the number of PC and the y-axis represents the standard deviation. The optimal number of principal components appears at the inflection point and was marked by a red arrow. Figure S2: Survival analysis for validation data. The survival curve is shown above, with the x-axis representing the survival time (days), the y-axis representing the survival probability, and the censored values are indicated by “+” in the curve. Below is the risk table showing the number (or percentage) of survivors at each time point. Figure S3. PPI network of key genes of the branch point. The nodes represent genes and the edges represent interaction relationships. Node colors represent gene sets, consistent with Figure 3. Figure S4. PPI networks of cell cluster-specific genes. The nodes represent genes and the edges represent interaction relationships. Node colors represent the auroc of genes generated by SC3 and the legend is shown below. Table S1: The detailed information of patients. Table S2: Key genes which promote the occurrence of the branch in cell trajectory. Table S3: Background genes of stage III for cibersort. Table S4: CIBERSORT result of patients in stage III. Table S5: literature validation for targets. Table S6: In this table, the drug-target relationship is represented as 0-1, and 1 represents that the gene is the target of the drug. Table S7: Marker genes from SC3. Table S8: drug-target-cluster relationships. Table S9: This table shows the literature validation results of CCLE intersection drugs. Only drugs that have not shown efficacy (logFC > 0) on OSCC from the experimental data of CCLE were verified, and drugs that already have been proved effective (logFC < 0) were marked in green color.
