# Supplementary material for: Exploring Precise Medication Strategies for OSCC Based on Single-Cell Transcriptome Analysis from a Dynamic Perspective
Source: Cancers (Basel). 2022 Sep 30;14(19):4801. doi: 10.3390/cancers14194801 (PMC9564072; doi:10.3390/cancers14194801)

figure S4. PPI networks of cell cluster-specific genes. The nodes represent genes and the edges represent interaction relationships. Node colors represent the auroc of genes generated by SC3 and the legend is shown below.

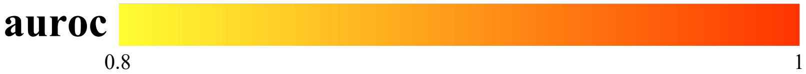

Cell cluster 1

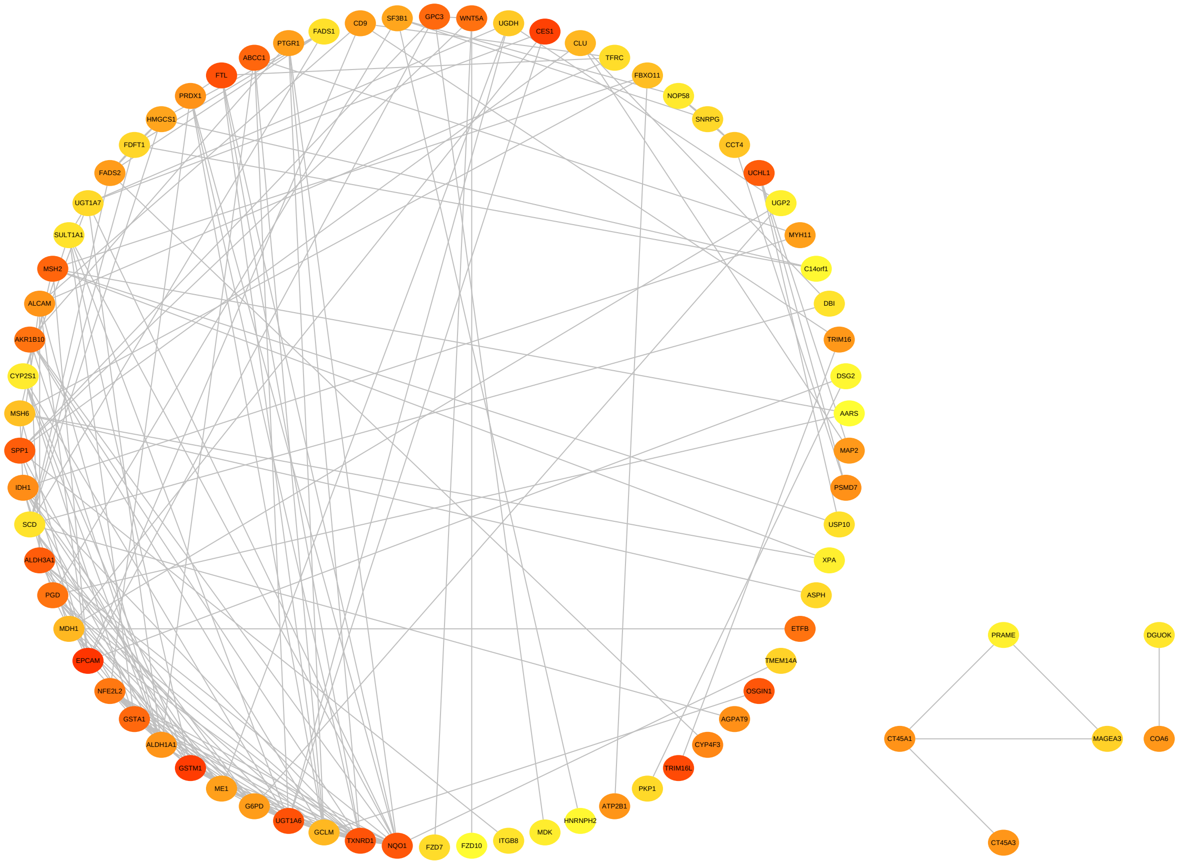

Cell cluster 2

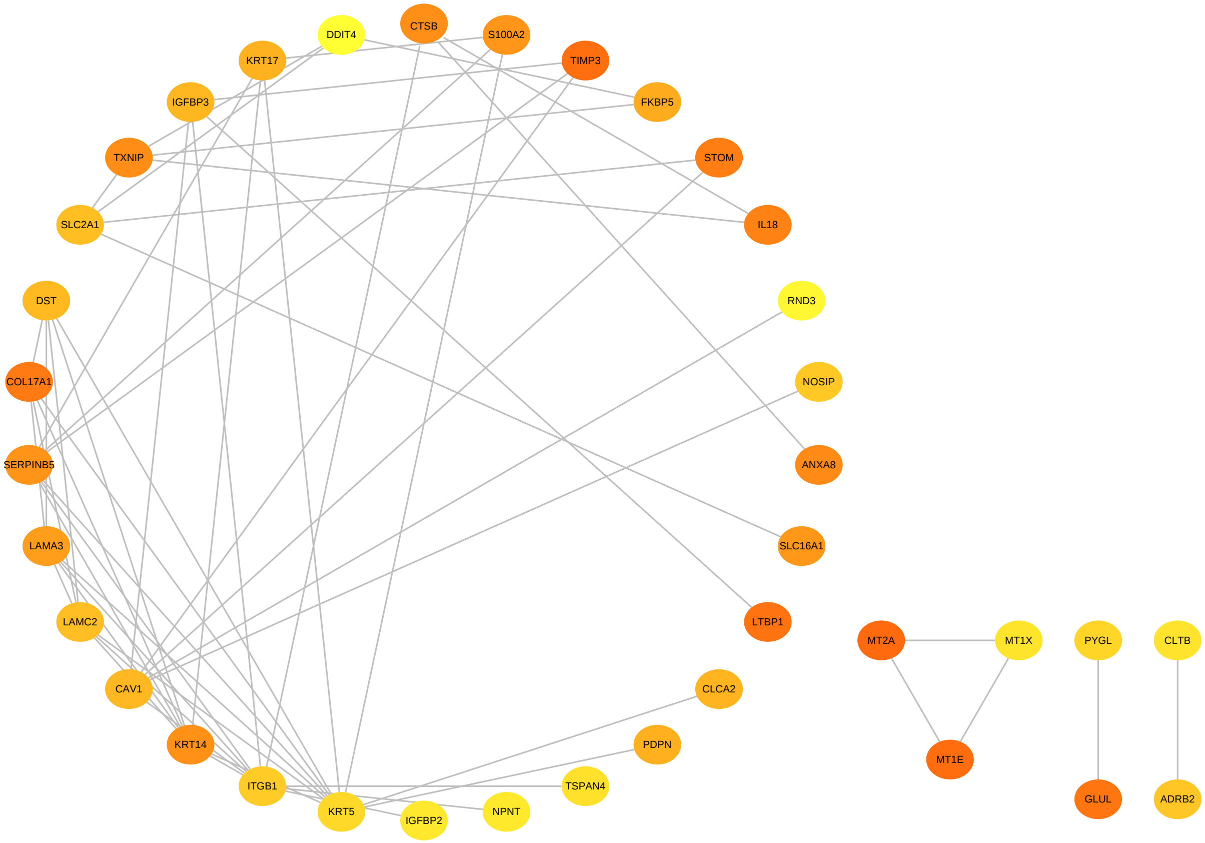

Cell cluster 3

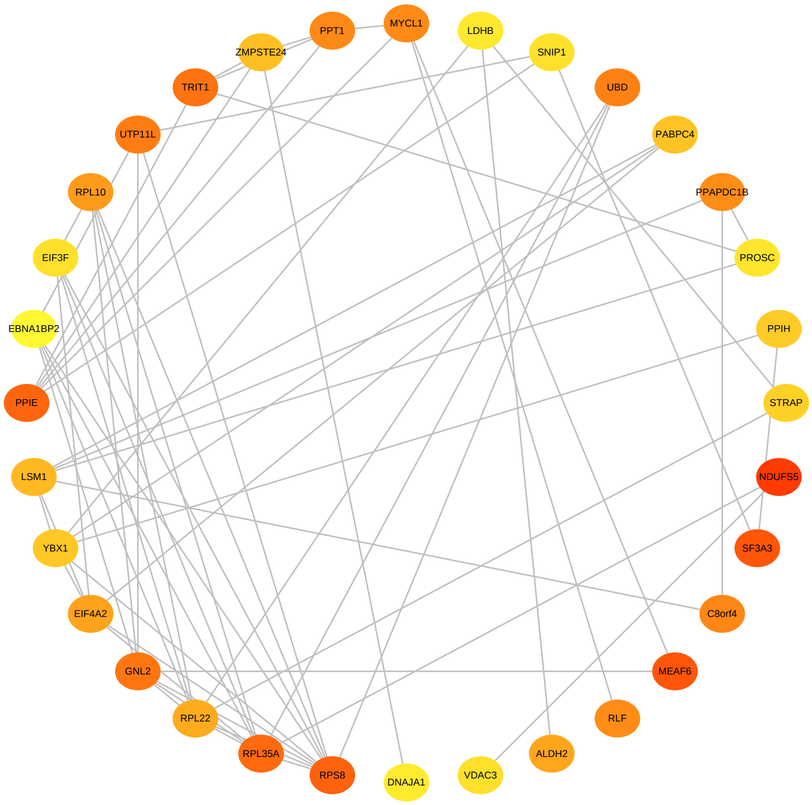

Cell cluster 4

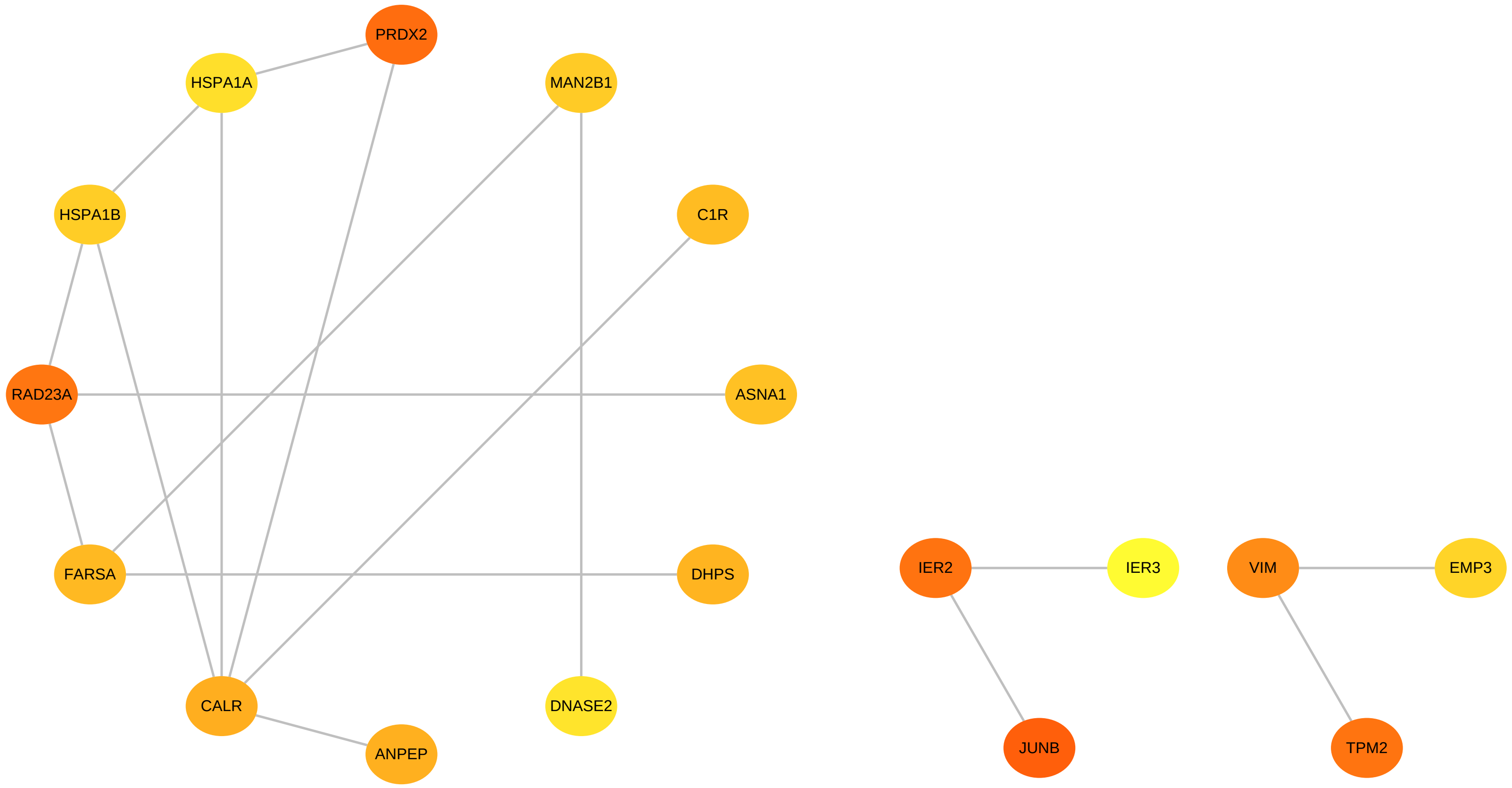

Cell cluster 5

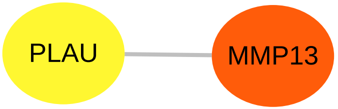

Cell cluster 6

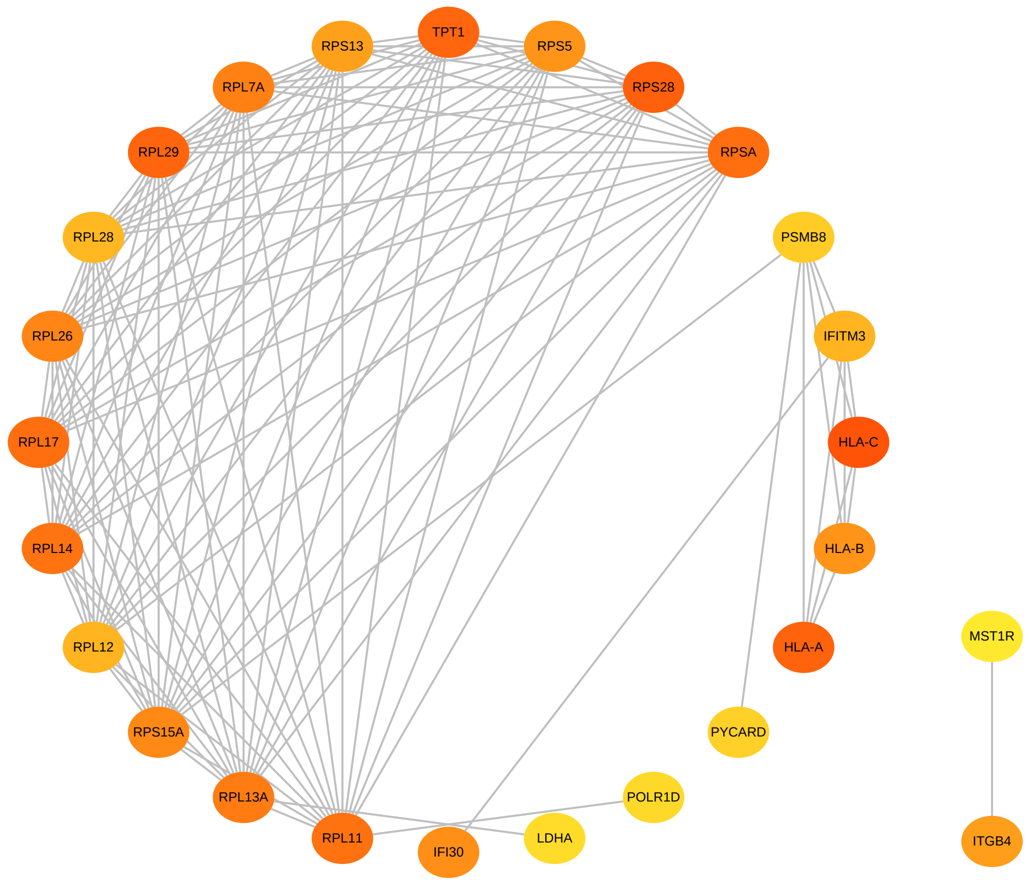

Cell cluster 7

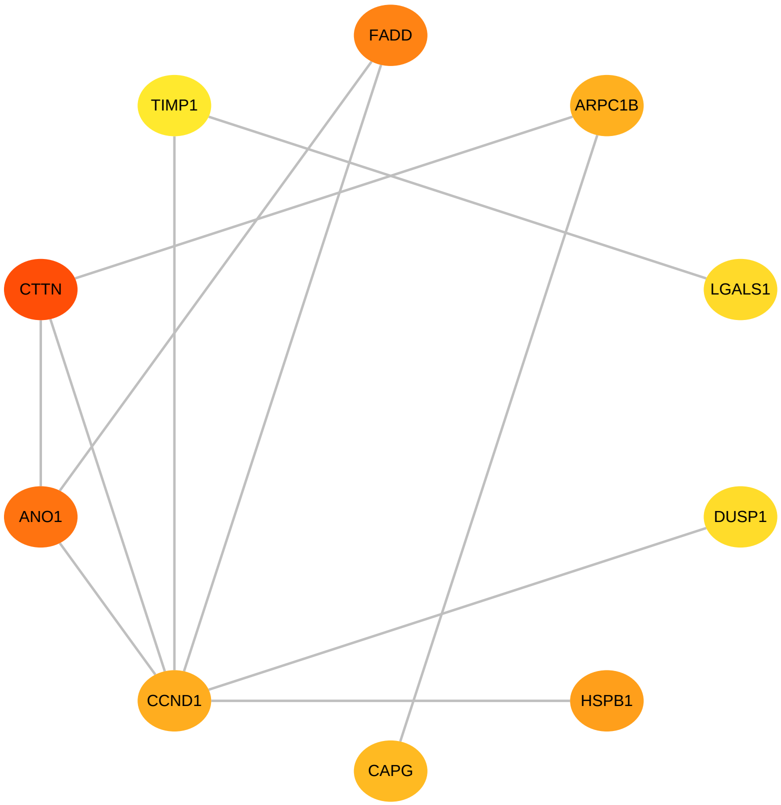

Cell cluster 8

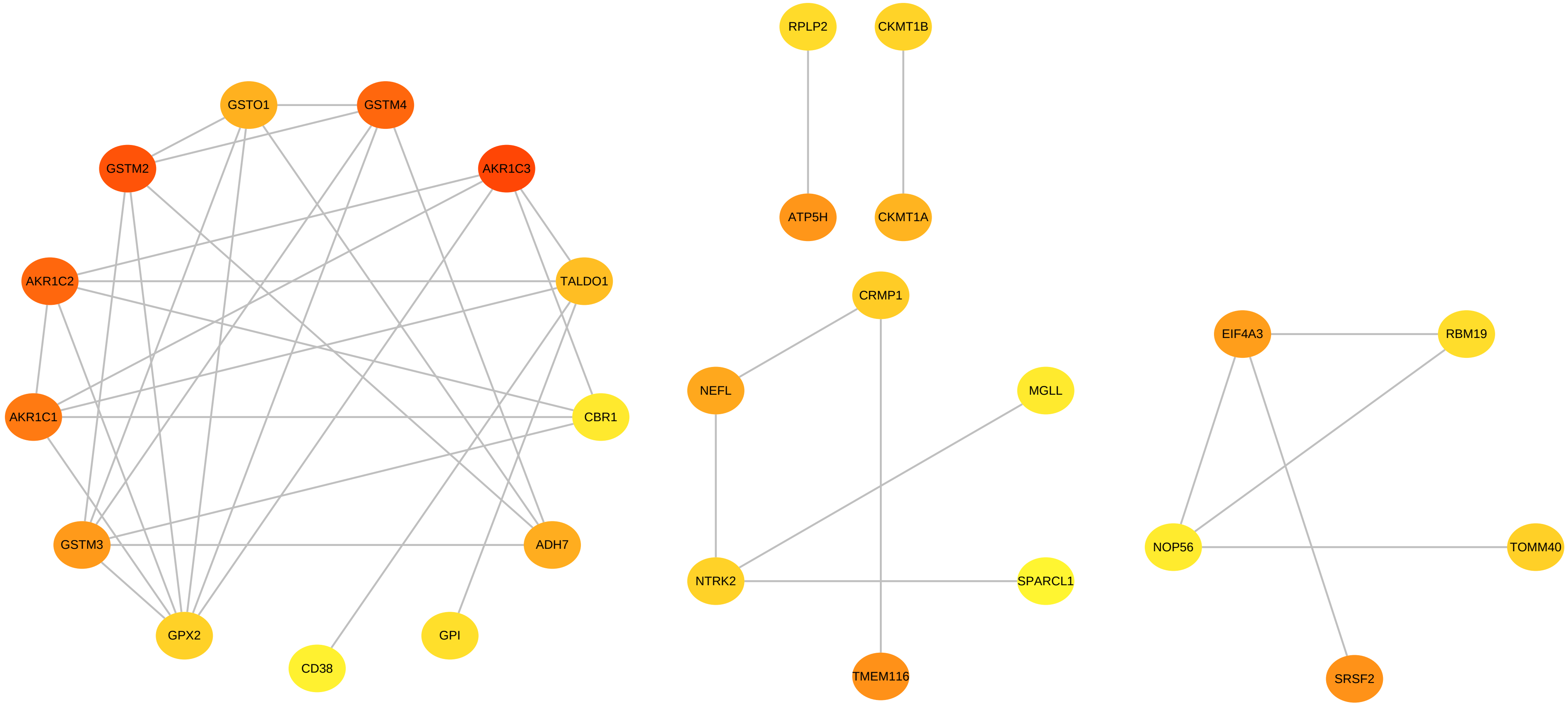

Cell cluster 9

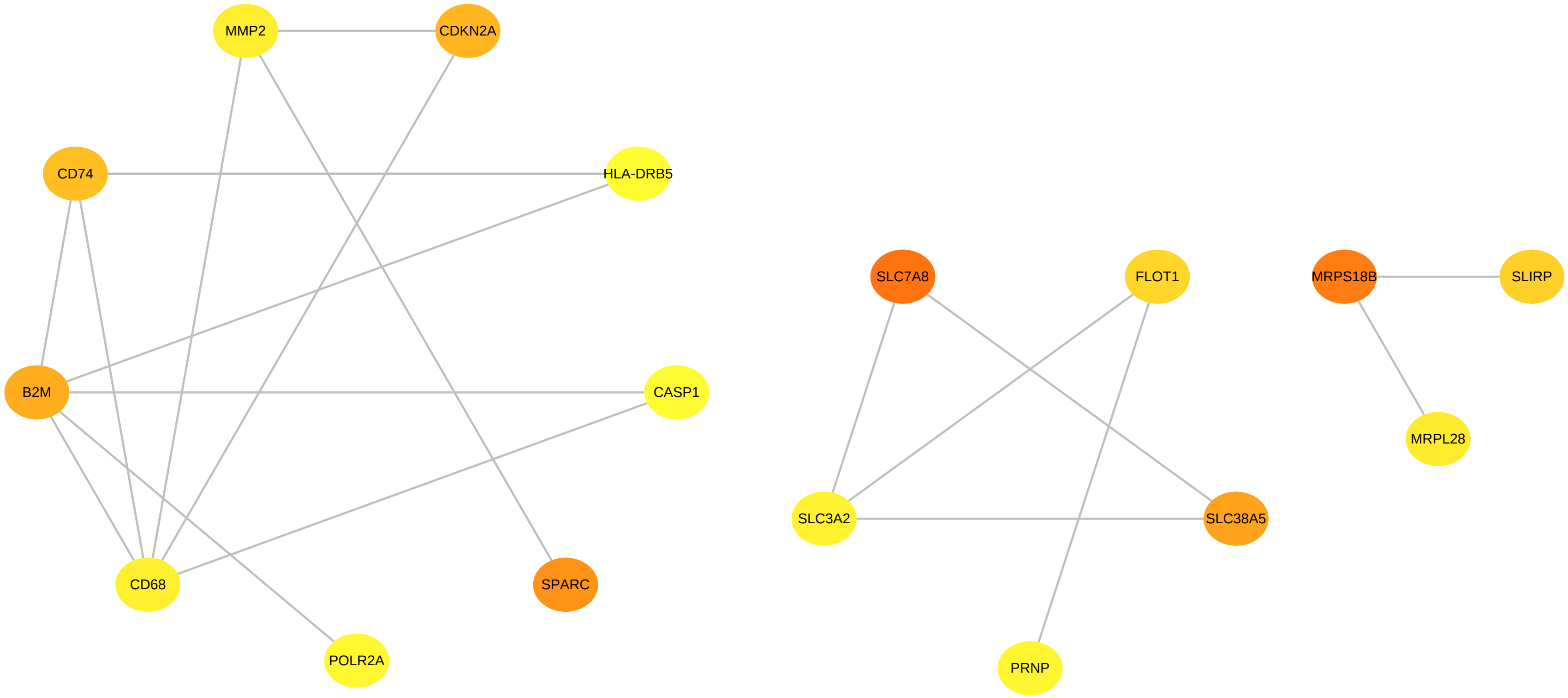

Cell cluster 10

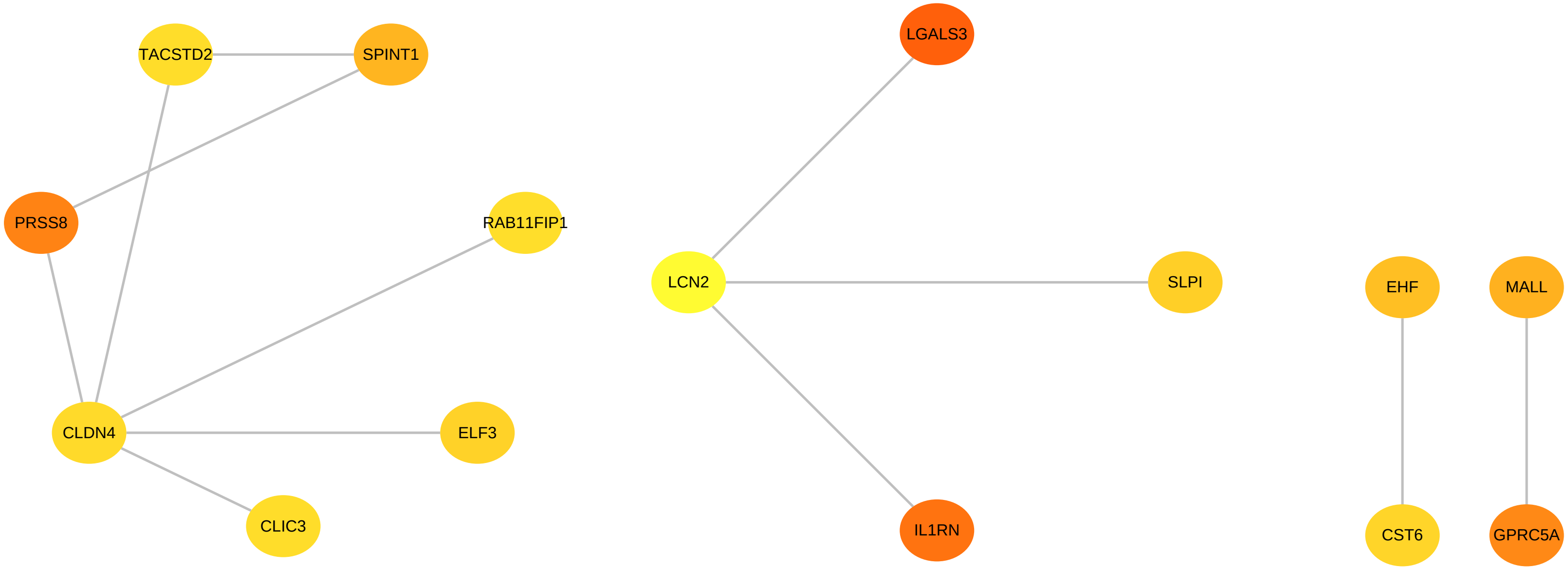

Supplement: Supplementary file 1 [file cancers-14-04801-s001.zip › figure S4.pdf]
