# Supplementary figures and images for: Exploring Precise Medication Strategies for OSCC Based on Single-Cell Transcriptome Analysis from a Dynamic Perspective
Source: Cancers (Basel). 2022 Sep 30;14(19):4801. doi: 10.3390/cancers14194801 (PMC9564072; doi:10.3390/cancers14194801)

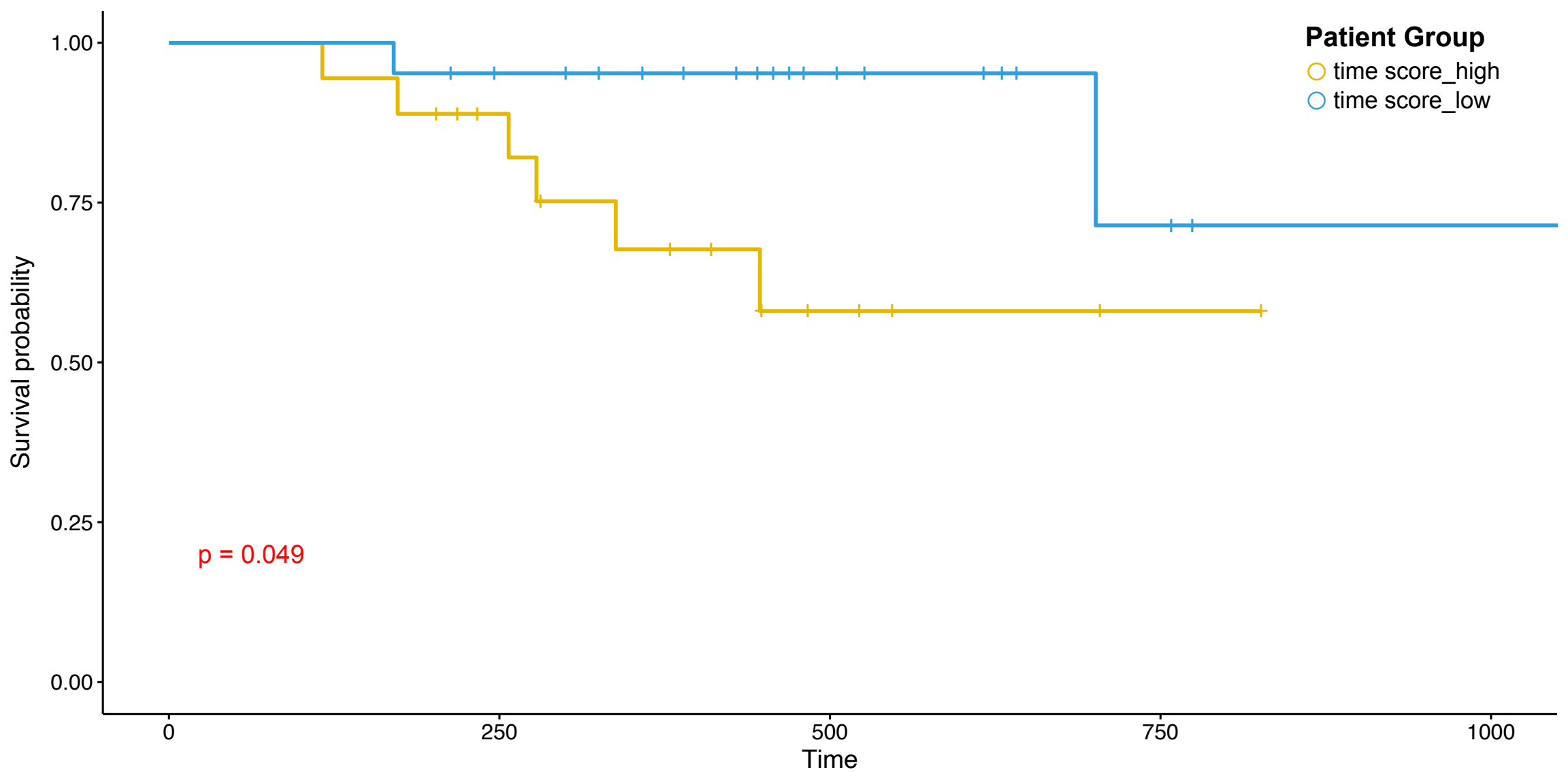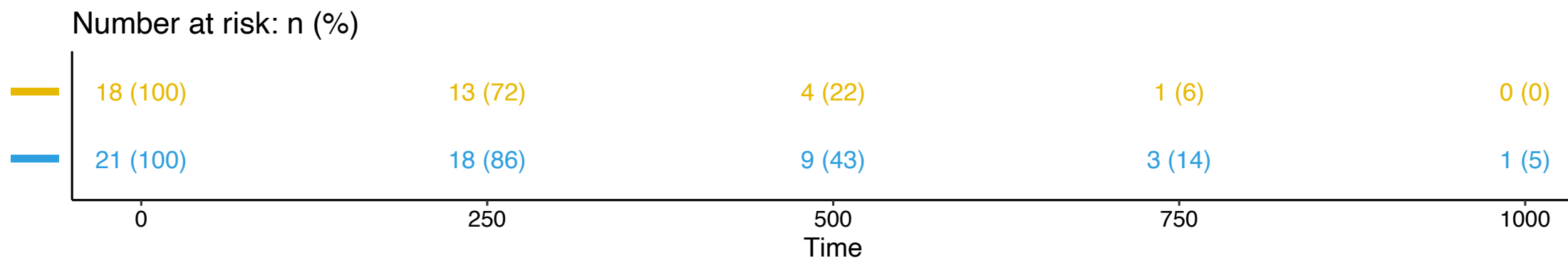

Supplement: Supplementary file 1 [file cancers-14-04801-s001.zip › figure S2.pdf]
